# Unsaturation of vapour pressure inside leaves of two conifer species

**DOI:** 10.1038/s41598-018-25838-2

**Published:** 2018-05-16

**Authors:** Lucas A. Cernusak, Nerea Ubierna, Michael W. Jenkins, Steven R. Garrity, Thom Rahn, Heath H. Powers, David T. Hanson, Sanna Sevanto, Suan Chin Wong, Nate G. McDowell, Graham D. Farquhar

**Affiliations:** 1 College of Science and Engineering, James Cook University, Cairns, Queensland Australia; 2 Research School of Biology, The Australian National University, Canberra, Australian Capital Territory Australia; 3 Department of Ecology and Evolutionary Biology, University of California Santa Cruz, California, USA; 4 METER Group, Inc. Pullman, Washington, USA; 5 Earth and Environmental Sciences Division, Los Alamos National Laboratory, Los Alamos, New Mexico USA; 6 Department of Biology, University of New Mexico, Albuquerque, New Mexico USA; 7 Earth Systems Analysis and Modelling Group, Pacific Northwest National Laboratory Richland, Washington, USA

## Abstract

Stomatal conductance (*g*_s_) impacts both photosynthesis and transpiration, and is therefore fundamental to the global carbon and water cycles, food production, and ecosystem services. Mathematical models provide the primary means of analysing this important leaf gas exchange parameter. A nearly universal assumption in such models is that the vapour pressure inside leaves (*e*_i_) remains saturated under all conditions. The validity of this assumption has not been well tested, because so far *e*_i_ cannot be measured directly. Here, we test this assumption using a novel technique, based on coupled measurements of leaf gas exchange and the stable isotope compositions of CO_2_ and water vapour passing over the leaf. We applied this technique to mature individuals of two semiarid conifer species. In both species, *e*_i_ routinely dropped below saturation when leaves were exposed to moderate to high air vapour pressure deficits. Typical values of relative humidity in the intercellular air spaces were as low 0.9 in *Juniperus monosperma* and 0.8 in *Pinus edulis*. These departures of *e*_i_ from saturation caused significant biases in calculations of *g*_s_ and the intercellular CO_2_ concentration. Our results refute the longstanding assumption of saturated vapour pressure in plant leaves under all conditions.

## Introduction

Stomata are microscopic pores that mediate the uptake of CO_2_ and loss of water from terrestrial plant leaves^1^. Analyses of stomatal function were greatly facilitated by the development of a method for continuous, non-destructive quantification of stomatal conductance (*g*_s_), the Gaastra method^2^. The key to this method is to assume that air inside the leaf is saturated with water vapour, with the saturation vapour pressure (*e*_s_) then calculated according to an exponential relationship with leaf temperature (*T*_l_). With this assumption, *g*_*s*_ can be calculated from measurements of the transpiration rate (*E*), the vapour pressure of the air outside the leaf (*e*_a_), and *T*_l_, assuming boundary layer conductance (*g*_b_) is known^2^. Furthermore, once *g*_s_ has been established, the intercellular CO_2_ concentration (*c*_*i*_) can be calculated^3,4^. Knowing *c*_i_ is useful for relating photosynthetic metabolism and water-use efficiency to environmental conditions. The Gaastra^2^ method of quantifying *g*_s_ and *c*_i_ has become standard practice in leaf gas exchange studies and is employed in all commercial gas exchange systems.

It has become a dogmatic assumption in the field of plant physiology that the intercellular vapour pressure (*e*_i_) is saturated. If *e*_i_ becomes unsaturated under some conditions, this will cause a bias in estimations of *g*_s_ and *c*_i_. The vapour pressure inside leaves cannot be measured directly, but in a few studies indirect techniques have been applied to address the question. Results have been mixed, with some authors finding evidence of unsaturation^5–9^, and others no such evidence^10–12^; thus, the question has remained unresolved for decades.

**Figure 1:**
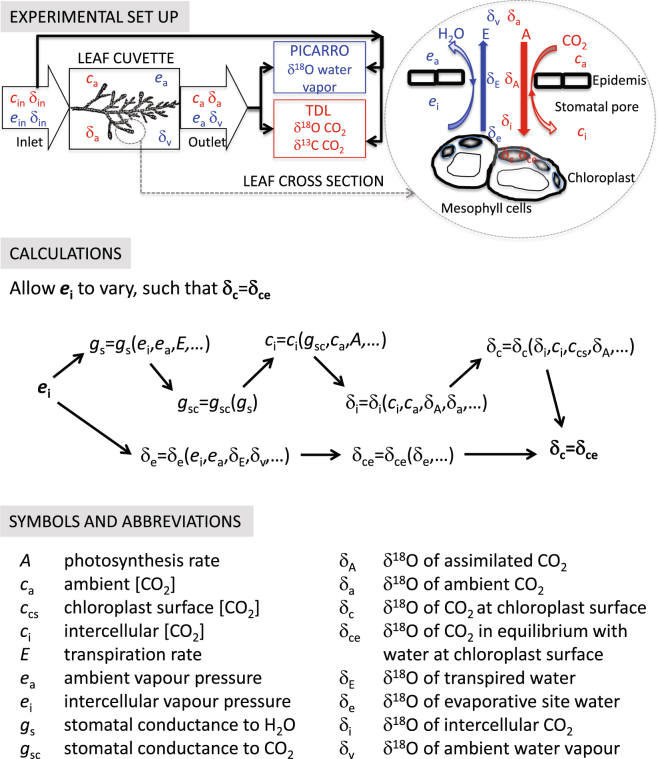
Experimental design underlying our method for estimating the intercellular vapour pressure, *e*_i_. A twig was placed in the leaf cuvette of a Li-Cor 6400 portable photosynthesis system. The flow of air in and out of the cuvette was split and diverted to water vapour and CO_2_ spectroscopic isotope analysers. In the top panel, symbols and fluxes in red relate to CO_2_ and those in blue to water vapour. The middle panel shows the basic flow of calculations, and the bottom panel provides definitions for symbols and abbreviations.

Here we present results from a new type of experiment aimed at quantifying *e*_i_ (Fig. 1). The underlying theory and the accompanying system of equations are described in full in the Supplementary Material. As air passes over a C_3_ leaf, CO_2_ diffuses through the stomata into the intercellular air space, and from there into the chloroplast, where some of it is fixed by RuBisCO. During diffusion through liquid, CO_2_ can exchange its oxygen atoms with those in water, with the rate of exchange greatly accelerated by the enzyme carbonic anhydrase. The most relevant site for carbonic anhydrase in this diffusion pathway is thought to be at the chloroplast surface^13^. Of the CO_2_ that exchanges its oxygen atoms with water at the chloroplast surface, not all will be fixed by RuBisCO, and some will diffuse back to the intercellular air space. Therefore, the intercellular air space contains a mixture of CO_2_ that has diffused in from the atmosphere carrying δ^18^O signature δ_a_, and CO_2_ that has diffused back from the chloroplast surface carrying δ^18^O signature δ_c_. We use the symbol δ_i_ to refer to the δ^18^O of this CO_2_ mixture in the intercellular air space.

Water at the evaporative sites of leaves becomes enriched in ^18^O during transpiration^14^, with the extent of enrichment described by the well-known Craig-Gordon equation^15^. Because chloroplasts are appressed against the cell walls lining the intercellular air spaces in C_3_ plants^16^, it can be assumed that the δ^18^O of water at the chloroplast surface is very close to that at the evaporative sites^17^. The resulting enrichment of ^18^O in water at the chloroplast surface sets up a catena of enrichment of δ^18^O in CO_2_, with that at the chloroplast surface being highest, that in the intercellular air spaces intermediate, and that in the ambient air lowest. Thus, under typical conditions, we expect to find the pattern, δ_c_ > δ_i_ > δ_a_.

We combined measurements of the oxygen isotope composition of CO_2_ entering and exiting a leaf gas exchange cuvette with gas exchange parameters to estimate δ_i_. This estimate of δ_i_ is sensitive to *c*_i_, and is therefore sensitive to *g*_s_, which is in turn sensitive to the assumed value of *e*_i_ (Fig. 1). From concurrent measurements of the δ^18^O of transpired water, we simultaneously estimated δ^18^O of water at the evaporative sites lining the intercellular air spaces (δ_e_). The δ_e_ is also sensitive to the assumed value of *e*_i_, but typically much less so than δ_i_. We then calculated the δ^18^O of CO_2_ in equilibrium with δ_e_, which we term δ_ce_. Keeping in mind that the true δ_i_ must reflect a mixture between δ_ce_ and δ_a_, we then increased the air vapour pressure deficit (*D*), to see if the apparent δ_i_, calculated by assuming saturated *e*_i_, would remain bounded between δ_ce_ and δ_a_. Here and throughout the manuscript we present *D* as the difference between the saturation vapour pressure at air temperature (*e*_s(*T*a)_) and the air vapour pressure (*e*_a_). We prefer this formulation of *D* in this context because it provides a description of the evaporative demand of the air outside the leaf which does not depend on an assumed value of *e*_i_.

If δ_i_ exceeds δ_ce_ when δ_a_ is well below δ_ce_, this indicates an error in the calculation of δ_i_. Unsaturation of *e*_i_ can cause this error. In the next step of our analysis, we solved for the *e*_i_ that would be required for δ_c_ (calculated from δ_i_) to be equal to δ_ce_. This allowed us to quantitatively estimate *e*_i_ over a range of *D*. We used these estimates of *e*_i_ to test the longstanding assumption that the vapour pressure of air inside leaves remains saturated even as the evaporative demand of the air outside the leaf increases.

## Results and Discussion

**Figure 2:**
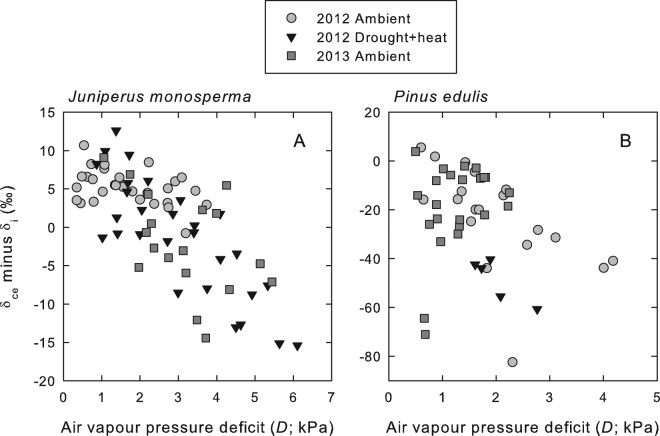
The difference between the δ^18^O of CO_2_ in equilibrium with evaporative site water (δ_ce_) and the δ^18^O of CO_2_ in the intercellular air spaces (δ_i_) plotted as a function of the air vapour pressure deficit (*D*) to which the leaf was exposed for *J. monosperma* (**A**) and *P. edulis* (**B**). The δ_i_ and δ_ce_ were calculated assuming saturation of vapour pressure in the intercellular air spaces. Negative values of δ_ce_-δ_i_ are inconsistent with theoretical expectations in this context, and indicate that the assumption of saturation of intercellular vapour pressure, *e*_i_, was invalid.

The δ_a_ in our experiment, measured in the gas stream exiting the gas exchange cuvette, ranged from 12 to 27‰ (VSMOW). The δ_ce_ ranged from 40 to 68‰ for *J. monosperma* and from 52 to 71‰ for *P. edulis*. Thus, as expected, δ_ce_ was always substantially higher than δ_a_. As described above, theory dictates that δ_i_ should lie between δ_a_ and δ_ce_. However, as *D* increased in the cuvette, we observed that the apparent δ_i_ became larger than δ_ce_, such that the difference between δ_ce_ and δ_i_ became negative in both species (Fig. 2). Calculation of both parameters assumed saturation of *e*_i_. The increasingly negative values of δ_ce_-δ_i_ with increasing *D* indicate errors in the estimation of δ_i_ under high *D*; these errors can be reconciled by allowing *e*_i_ to drop below saturation as *D* increased.

Are there other possible explanations for δ_i_ becoming larger than δ_ce_ at moderate to high *D*? We considered five possible alternative explanations: (1) decreasing effectiveness of carbonic anhydrase with decreasing leaf water potential, such that the δ^18^O of CO_2_ at the chloroplast surface might not be completely equilibrated with local water; (2) somewhat less ^18^O-enriched water at the chloroplast surface than that at the evaporative sites due to a Péclet effect^18^; (3) a fractionation factor for static diffusion of H_2_^18^O through the stomatal pore of 32‰ rather than 28‰^19–21^; (4) an error associated with neglecting cuticular conductance in gas exchange calculations^22,23^; and (5) a bias in the measurement of *T*_l_ by the energy balance method. These possible alternative explanations are addressed in full in the Supplementary Material. In summary, none of them can account satisfactorily for observations of δ_i_ surpassing δ_ce_. Therefore, the most likely explanation remains that *e*_i_ declined below saturation as *D* increased.

**Figure 3:**
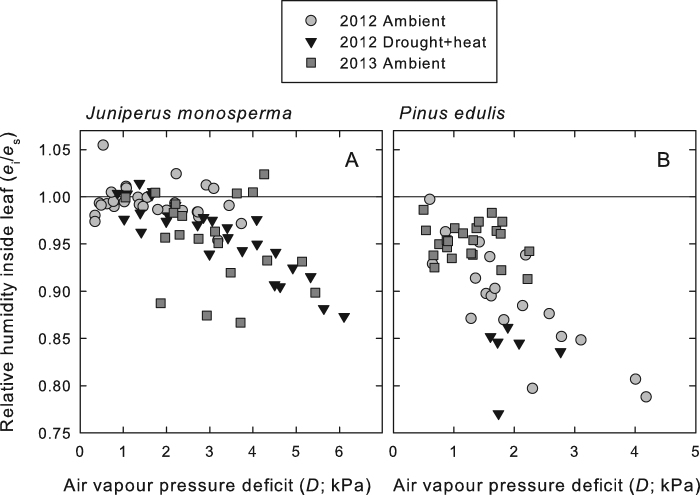
Relative humidity in the intercellular air spaces inside the leaves of two semiarid conifer species plotted as a function of the air vapour pressure deficit (*D*) to which the leaves were exposed in the gas exchange cuvette for *J. monosperma* (**A**) and *P. edulis* (**B**). The relative humidity is defined as *e*_i_/*e*_s_, where *e*_i_ is the intercellular vapour pressure and *e*_s_ is the saturation vapour pressure at leaf temperature. A relative humidity of unity indicates saturation, and is denoted by the horizontal line within each panel. The analysis demonstrates clear evidence of unsaturation of the internal humidity, even at rather modest air vapour pressure deficits, for these semiarid conifers. A segmented regression analysis indicated a breakpoint in the regression for *J. monosperma* at air vapour pressure deficit of 1.6 kPa, below which the slope was not significant. Above 1.6 kPa for *J. monosperma*, the slope was estimated to be −0.021 kPa^−1^ (*R*^2^ = 0.35, *P* < 0.0001, *n* = 54). For *P. edulis*, no breakpoint was identified, and a slope of −0.049 kPa^−1^ was estimated (*R*^2^ = 0.49, *P* < 0.0001, *n* = 48).

**Figure 4:**
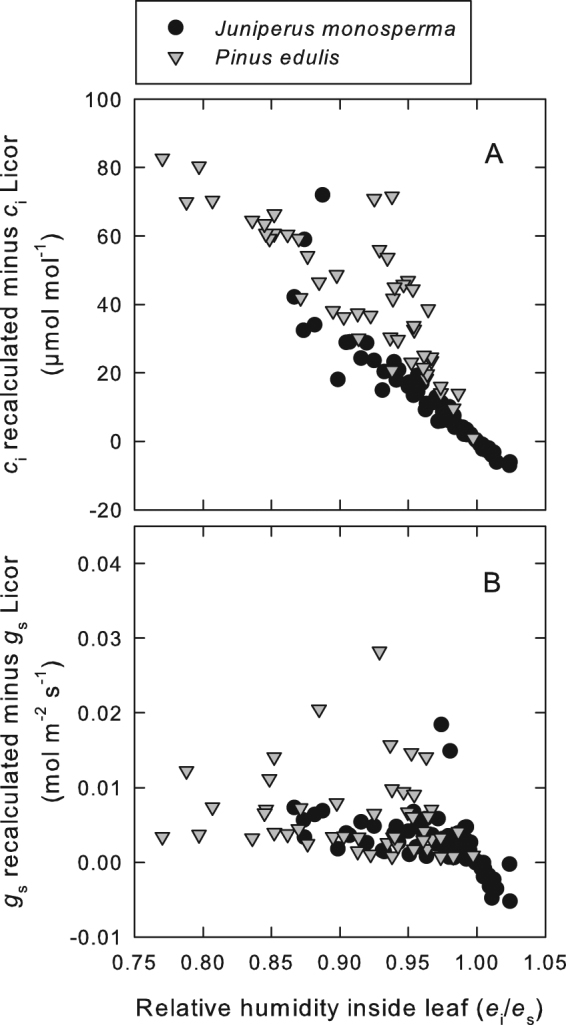
The difference between the intercellular CO_2_ concentration (*c*_i_) calculated without assuming saturation of intercellular vapour pressure (*e*_i_) and that calculated by the Li-Cor portable photosynthesis system assuming saturation of *e*_i_ (**A**) and the difference between stomatal conductance (*g*_s_) calculated without assuming saturation of *e*_i_ and that calculated by the Li-Cor portable photosynthesis system assuming saturation of *e*_i_ (**B**) plotted against the relative humidity in the intercellular air spaces. The relative humidity inside the leaf on the x-axis was generated from calculations that did not assume saturation of *e*_i_.

Next, we solved for the *e*_i_ required for δ_c_, the δ^18^O of CO_2_ at the chloroplast surface calculated from δ_i_, to be equal to δ_ce_, the δ^18^O of CO_2_ at the chloroplast surface calculated from δ_e_. This allowed us to quantitatively estimate *e*_i_. This calculation required an estimate of *g*_mc_, the conductance to CO_2_ from the intercellular air space to the site of carbonic anhydrase activity. We inferred values of *g*_mc_ such that they resulted in estimates of *e*_i_ near to saturation when *D* was lowest. Estimates of the relative humidity inside the leaf made by assuming these values of *g*_mc_ are shown in Fig. 3. These estimates decreased as *D* increased in both species, declining to values in the range of 0.9 in *J. monosperma* and 0.8 in *P. edulis*. As a result of these departures of *e*_i_ from saturation, our analysis indicated that *c*_i_ could be underestimated by as much as 80 µmol mol^−1^, and *g*_s_ by as much as 30 mmol m^−2^ s^−1^ (Fig. 4).

To demonstrate the impact of choosing different values for *g*_mc_, we conducted a sensitivity analysis, in which we calculated the relative humidity inside the leaf (*e*_i_/*e*_s_) for values of *g*_mc_ twice those originally assigned and for values half those originally assigned. These estimates of *e*_i_/*e*_s_ are shown in Supplementary Figure 1. From this figure, one can see that doubling the assigned *g*_mc_ shifted the range of *e*_i_/*e*_s_ estimates up, and halving it shifted the range of *e*_i_/*e*_s_ estimates down. However the shifts were not so large as to substantially alter our interpretation of the results. Thus, while it is clear that estimates of *e*_i_/*e*_s_ by our technique are sensitive to assigned values of *g*_mc_, they are not hypersensitive, and our conclusion that *e*_i_/*e*_s_ declined well below unity at moderate to high *D* would hold for any of the three parameterisations shown in Fig. 3 and Supplementary Figure 1.

Our observations of unsaturation of *e*_i_ at moderate to high *D* are in agreement with recent results from an experiment with angiosperm species *Gossypium hirsutum* and *Eucalyptus pauciflora* (Wong, Canny and Farquhar, unpublished). In that experiment, gas exchange was measured independently on upper and lower leaf surfaces, with the lower leaf surface exposed to air with CO_2_ concentration reduced so that net photosynthetic rate was zero, and the upper leaf surface to air with CO_2_ concentration near the ambient atmospheric value. Under such conditions, a gradient of *c*_i_ should have existed from the upper to the lower surface. As *D* increased above about 2 kPa, the apparent *c*_i_ gradient reversed, indicating that the calculations of *c*_i_ were in error; the most parsimonious explanation was unsaturation of *e*_i_.

In our experiment, *P. edulis* showed a stronger tendency toward unsaturation of *e*_i_ with increasing *D* than did *J. monosperma*. The two species are known to differ in their hydraulic behaviour: *P. edulis* is relatively isohydric, whereas *J. monosperma* is relatively anisohydric^24,25^; stem hydraulic conductivity decreases more strongly in response to decreasing soil water potential in *P. edulis* than in *J. monosperma*^26^; and the bulk modulus of elasticity is lower in *P. edulis* than in *J. monosperma* leaf tissue^25^, suggesting mesophyll cell wall function differs between the two species. Our data suggest a coordination in hydraulic behaviour between the stem xylem and the mesophyll cell walls. As the evaporation rate from the mesophyll cell walls increased in response to increasing *D*, stronger flexure of the menisci in the cell wall pores would have decreased the cross sectional area of pore space, causing an increase in frictional resistance to water movement, and therefore a reduction in mesophyll cell wall hydraulic conductivity^27^. Our results suggested a steeper decline in mesophyll cell wall hydraulic conductivity in *P. edulis* than in *J. mosoperma* as *D* increased, analogous to steeper declines in xylem hydraulic conductivity with decreasing water availability.

Here we provide the first experimental evidence of unsaturation of *e*_i_ in conifer trees by applying a novel method to estimate *e*_i_ under field conditions. There are few previous reports of unsaturation of *e*_i_, likely because there is no simple method for directly measuring *e*_i_. Recent theoretical treatments differ in their assertions as to the importance of unsaturation of *e*_i_ for leaf gas exchange^28,29^. We show here that the potential errors that can occur in the estimation of *g*_s_ and *c*_i_ by assuming saturation of *e*_i_ can be significant. Leaf gas exchange measurements are globally common, and errors associated with assuming saturation of *e*_i_ could have a major impact on their interpretation. Our method of determining *e*_i_ has potential for broad application, given recent technological advances in laser-based methods for determining δ^18^O of CO_2_ and water vapour^30^. This could open the door to new insights into how leaves regulate water loss, with fundamental implications for understanding environmental constraints on plant function.

## Methods

Our experiment took place at Los Alamos National Laboratory’s SUrvival-MOrtality (SUMO) outdoor experiment (35.8180°N, 106.3053°E, elevation 2180 m). The soil texture at the site is sandy loam at the surface grading to a clay loam with depth^31,32^. Soil depth ranges from 40 to 80 cm. The site is located in an upland topographic position near the ecotone between piñon-juniper woodland and ponderosa pine forest. *Pinus edulis* and *Juniperus monosperma* are the dominant tree species. The 30 year mean annual temperature and precipitation at a meteorological tower located about 1 km from the site are 9.2 °C and 470 mm, respectively. Roughly half of the annual total precipitation falls from July to September during the North American Monsoon.

For this study, we used mature trees of both species, located within and on the periphery of the SUMO experiment. The experiment comprises control, drought and heat treatments designed to mimic historic conditions during mortality-inducing drought in piñon-juniper woodlands^33^ and during extreme heat waves. The control trees were growing in ambient temperature and precipitation with no experimental manipulation; and the treated trees were exposed to ~50% precipitation reduction by rainfall exclusion and ~5 °C above ambient temperature by open-top chambers^34^. The rainfall exclusion structure was installed on 1 June 2012 and heat treatments were operational on 11 June 2012^35^.

Our measurements took place from 11–24 September 2012 and from 23–30 August 2013, and included individuals of both *J. monosperma* and *P. edulis*. Control trees of both species were measured in both campaigns and drought + heat trees were measured during the 2012 campaign. Ambient conditions were drier during the 2013 campaign and gas exchange rates in drought + heat trees were too low for measurements at that time, so the 2013 campaign included only un-manipulated control trees.

We coupled a Tunable Diode Laser (TDL; TGA100A, Campbell Scientific Inc., Logan, UT, USA) to a portable photosynthesis system (Li-Cor 6400; Li-Cor Biosciences, Lincoln, NE, USA) fitted with a conifer cuvette (Li-Cor 6400-22) to quantify the concentration of CO_2_ and its isotopic composition (δ^13^C and δ^18^O) in gas entering and exiting the leaf chamber. The gas streams were plumbed directly into the TDL using ultra-low porosity tubing (Synflex type 1300 1/4 in diameter; Saint Gobain Performance Plastics, Northboro, MA, USA). The TDL data acquisition and processing were as described previously^36^.

Calibration of the TDL was maintained by using two working standard (WS) calibration tanks during measurements. These WS tanks were calibrated against World Meteorological Organization (WMO) certified standard tanks. To account for instrument drift, the TDL measured the high and low WS tanks during a 3 min cycle also including measurements of the gas exchange cuvette inlet and outlet gas streams. For each 3 min cycle, we calculated the deviation between the measured values and the known values to determine a gain and offset for each isotopologue^37^. These gain and offset values were then applied to all data in the 3 min measurement cycle. The measurements of gas entering and exiting the cuvette fell within the range of isotopologue concentrations within the two WS tanks.

Before the gas streams entered the TDL, part of the flow was diverted to a cavity-ring-down spectroscopy water isotope analyser (Picarro L2130-i, Picarro Inc., Santa Clara, CA, USA) that measured the δ^18^O of water vapour. The pre- and post-cuvette gas streams were measured for 10 min each at approximately 1 Hz, and the final 5 min of measurements was averaged for each gas stream. The water isotope analyser was calibrated with WS waters. These were introduced into the analyser either using the associated vaporizer unit or by sampling air from sealed plastic bags equilibrated with WS water samples enclosed within them. The temperature dependent liquid-vapour equilibrium fractionation factor was applied in the latter case. The WS water vapours were run once per day.

Our sampling regime was designed to loosely mimic the increasing *D* that a leaf typically experiences from early morning through to the afternoon. For the most part, we measured one foliage sample per day. The terminal part of a *J. monosperma* or *P. edulis* twig was placed in the Licor conifer chamber one to two hours after sunrise. The entry point of the twig into the cuvette and all exposed gasket surfaces were covered with flexible putty (Terostat IX, Henkel Technologies, Düsseldorf, Germany) to minimize diffusion leaks. The first measurement generally took place under irradiance of 300 µmol photons m^−2^ s^−1^, and at *T*_l_ between 15 and 20 °C. The leaves were allowed to stabilise their gas exchange in the cuvette for about 30 min before a measurement began. Thereafter, we recorded gas exchange, δ^18^O of CO_2_ and δ^18^O of water vapour for 20 min. The irradiance and cuvette temperature were then increased, such that a series of measurements was made for each foliage sample from low to high *D*. The range of *T*_l_ in the dataset was from 14.6 to 40.8 °C, and the range of photosynthetically active radiation from 300 to 2200 µmol photons m^−2^ s^−1^. Chamber flow rate was varied between 250 and 500 µmol s^−1^, with the aim of maintaining a [CO_2_] drawdown in the leaf chamber of at least 15 µmol mol^−1^. The [CO_2_] within the chamber was approximately 390 µmol mol^−1^. The series of measurements for each foliage sample usually continued until gas exchange diminished as a result of high *D* to such an extent that a 15 µmol mol^−1^ [CO_2_] drawdown between chamber inlet and outlet could not be achieved.

All gas exchange and isotopic calculations are described in detail in the Supplementary Material. Segmented regression analysis was performed using SegReg freeware (https://www.waterlog.info/segreg.htm), and all other regression analyses were performed in Systat 12 (Systat Software Inc., San Jose, CA, USA).

### Data availability

The datasets generated and analysed during the current study are available from the corresponding author on reasonable request.

## Electronic supplementary material

Supplementary Material
