# Supplementary material for: Unsaturation of vapour pressure inside leaves of two conifer species
Source: Sci Rep. 2018 May 16;8:7667. doi: 10.1038/s41598-018-25838-2 (PMC5955884; doi:10.1038/s41598-018-25838-2)
Supplement: Supplementary file 1 — Supplementary Material [file 41598_2018_25838_MOESM1_ESM.pdf]

Supplementary Material

Article title: Unsaturation of vapour pressure inside leaves of two conifer species

Authors: Lucas A. Cernusak, Nerea Ubierna, Michael W. Jenkins, Steven R. Garrity, Thom Rahn, Heath H. Powers, David T. Hanson, Sanna Sevanto, Suan Chin Wong, Nate G. McDowell, and Graham D. Farquhar

1. Calculation of e_i from $\delta^{18}O$

Here we describe theory underlying our method for estimating the intercellular vapour pressure (e_i) based on coupled measurements of gas exchange and the $\delta^{18}O$ of CO_2 and water vapour entering and exiting the leaf cuvette. The transpiration rate (E) can be related to the leaf-to-air vapour pressure difference (e_i-e_a) and total conductance (g_t) as[1],

$$E = g_t \frac{(e_i - e_a)}{P} + E\frac{\bar{e}}{P}, \qquad \text{(Eqn.1)}$$

where g_t is the combined conductance to water vapour of the stomata plus boundary layer, e_i is the intercellular vapour pressure, e_a is the atmospheric vapour pressure, P is atmospheric pressure, and $\bar{e}$ is $(e_i+e_a)/2$. The final term, $E\frac{\bar{e}}{P}$, is a ternary correction. Stomatal conductance (g_s) relates to g_t as,

$$g_s = \frac{1}{\frac{1}{g_t} - \frac{1}{g_b}}, \qquad \text{(Eqn. 2)}$$

where g_b is boundary layer conductance to water vapour. The transpiration rate, E, can be measured in a leaf gas exchange cuvette as[1]

$$E = \frac{u_{in}(w_{out} - w_{in})}{s(1 - w_{out})}, \qquad \text{(Eqn. 3)}$$

where u_{in} is the flow rate of air entering the cuvette, w_{in} and w_{out} are the water vapour mole fractions of air entering and leaving the cuvette, and s is the leaf area inside the cuvette. The term e_a/P from Eqn (1) is assumed equal to w_{out}. The g_s can then be calculated if e_i is known and P is measured. In standard gas exchange calculations, e_i is assumed equal to the saturated vapour pressure (e_s) at leaf temperature (T_l):

$$e_s = 0.61365e^{\left(\frac{17.502T_l}{240.97+T_l}\right)} . \qquad \text{(Eqn. 4)}$$

Here, e_s is given in kPa and T_l in °C. Making this assumption and assuming a value for g_b allows calculation of g_s from Eqns (1) and (2). In the method we developed, we did not assume that e_i=e_s, but rather solved for e_i iteratively, by setting δ_c=δ_{ce}. This is elaborated upon below.

The rate of CO_2 assimilation (A) can be determined by also measuring the CO_2 mole fraction entering (c_{in}) and leaving (c_{out}) the gas exchange cuvette[1]:

$$A = \frac{u_{in}}{s}\left[c_{in} - c_{out}\left(\frac{1-w_{in}}{1-w_{out}}\right)\right] . \qquad \text{(Eqn. 5)}$$

The A can further be expressed as a function of the conductance to CO_2 and the drawdown in CO_2 mole fraction between the atmosphere and the intercellular air spaces:

$$A = g_{tc}(c_a - c_i) - E\bar{c} , \qquad \text{(Eqn. 6)}$$

where g_{tc} is the combined conductance to CO_2 of the stomata plus boundary layer and $\bar{c}$ is defined as (c_a+c_i)/2. The c_a is assumed equal to c_{out}. The final term in Eqn (6) is a ternary correction that accounts for the influence of transpiration on the diffusion of CO_2 into the leaf. The stomatal conductance to CO_2 (g_{sc}) can be calculated as g_s/1.6 and the boundary layer conductance to CO_2 (g_{bc}) as g_b/1.37. The g_{tc} can then be calculated as,

$$g_{tc} = \frac{1}{\frac{1}{g_{sc}}+\frac{1}{g_{bc}}} . \qquad \text{(Eqn. 7)}$$

Eqn (6) can now be solved for c_i:

$$c_i = \frac{c_a - \frac{A}{g_{tc}} - \frac{Ec_a}{2g_{tc}}}{1+\frac{E}{2g_{tc}}} . \qquad \text{(Eqn. 8)}$$

The oxygen isotope composition of CO_2 taken up by photosynthesis (δ_A) can be calculated from measurements of the $\delta^{18}O$ of CO_2 entering and leaving the gas exchange cuvette when combined with measurements of total CO_2 entering and leaving[2-4]:

$$\Delta_A = \frac{\xi(\delta_a - \delta_{in})}{1+\delta_a - \xi(\delta_a - \delta_{in})} , \qquad \text{(Eqn. 9)}$$

$$\xi = \frac{c_{in}}{c_{in} - c_a} , \qquad \text{(Eqn. 10)}$$

$$\delta_A = \frac{\delta_a - \Delta_A}{1+\Delta_A} , \qquad \text{(Eqn. 11)}$$

where c_{in} and c_a are CO_2 mole fractions entering and leaving the gas exchange cuvette, expressed in Eqn (10) with respect to dry air, and δ_{in} and δ_a are $\delta^{18}O$ of CO_2 entering and leaving the cuvette.

After δ_A is obtained from Eqns. 9-11, the $\delta^{18}O$ of CO_2 in the intercellular air spaces (δ_i) can then be calculated[4,5]. We present this calculation in two steps below. Eqn (12) represents the calculation of δ_i without the ternary correction (δ_{io}), and Eqn (13) then applies this correction[5].

$$\delta_{io} = \delta_A\left(1-\frac{c_a}{c_i}\right)\alpha_{tc} + \frac{c_a}{c_i}(\delta_a - \bar{a}) + \bar{a}\,, \qquad \text{(Eqn. 12)}$$

$$\delta_i = \frac{\delta_{io}+t\left[\delta_A\left(\frac{c_a}{c_i}+1\right)-\delta_a\frac{c_a}{c_i}\right]}{1+t}\,, \qquad \text{(Eqn. 13)}$$

where $\bar{a}$ is the fractionation for combined $C^{18}OO$ diffusion across the boundary layer and the stomata, α_{tc} is defined as $1+\bar{a}$, and t is a ternary correction factor. The $\bar{a}$ is defined as

$$\bar{a} = \frac{a_b(c_a-c_s)+a_s(c_s-c_i)}{c_a-c_i}\,, \qquad \text{(Eqn. 14)}$$

where a_b is $C^{18}OO$ fractionation for diffusion across the boundary layer (5.8‰)[6], a_s is that for diffusion through stomata (8.8‰)[6], and c_s is the CO_2 mole fraction at the leaf surface calculated as $c_s=c_a-A/g_{bc}$. The t is defined as

$$t = \frac{\alpha_{tc}E}{2g_{tc}}\,. \qquad \text{(Eqn. 15)}$$

The $\delta^{18}O$ of CO_2 at the chloroplast surface (δ_c) can then be calculated as[4]

$$\delta_c = \delta_A\left(1-\frac{c_i}{c_{cs}}\right)\alpha_w + \frac{c_i}{c_{cs}}(\delta_i - a_w) + a_w\,, \qquad \text{(Eqn. 16)}$$

where a_w is the $C^{18}OO$ fractionation during diffusion through liquid water, taken as 0.8‰[6], and α_w is defined as $1+a_w$. The c_{cs} is the CO_2 concentration at the chloroplast surface.

The $\delta^{18}O$ of transpired water (δ_E) can be calculated from measurements of the $\delta^{18}O$ of water vapour entering and leaving the gas exchange cuvette, in combination with measurements of water vapour mole fractions[7]:

$$\delta_E = \frac{w_a\delta_v - w_{in}\delta_{in(v)}}{w_a-w_{in}} - \frac{w_a w_{in}(\delta_v-\delta_{in(v)})}{w_a-w_{in}}\,, \qquad \text{(Eqn. 17)}$$

where w_{in} and w_a are water vapour mole fractions of air entering and leaving the cuvette, and $\delta_{in(v)}$ and δ_v are $\delta^{18}O$ of water vapour entering and leaving the cuvette. The $\delta^{18}O$ of the liquid water at the evaporative sites in the leaf (δ_e) can then be calculated from the measurements of δ_E as[8],

$$\delta_e = (1+\varepsilon^+)\left[(1+\varepsilon_k)(1+\delta_E)\left(1-\frac{e_a}{e_i}\right)+\frac{e_a}{e_i}(1+\delta_v)\right]-1\,, \qquad \text{(Eqn. 18)}$$

where ε^+ is the equilibrium fractionation for the phase change from liquid to vapour, and ε_k is the kinetic fractionation for diffusion through the stomata and boundary layer. The ε^+ can be calculated as[9]

$$\varepsilon^+(‰) = 2.644 - 3.206\left(\frac{10^3}{273.15+T_l}\right) + 1.534\left(\frac{10^6}{(273.15+T_l)^2}\right), \qquad \text{(Eqn. 19)}$$

and the ε_k as[10]

$$\varepsilon_k(‰) = \frac{28\frac{1}{g_s}+19\frac{1}{g_b}}{\frac{1}{g_s}+\frac{1}{g_b}}\,. \qquad \text{(Eqn. 20)}$$

The T_l in Eqn (19) is leaf temperature in °C. The 28 and 19 in Eqn (20) are fractionation factors for $H_2^{18}O$ diffusion through the stomata and boundary layer, respectively, scaled to per mil[11]. These values have been taken as 32 and 21 more recently[12], but there is ongoing debate as to which are the correct values[13]. Implications of assuming one set or the other are addressed below. The δ_e provides an estimate of the $\delta^{18}O$ of liquid water near the chloroplast surface. The fractionation between this water and CO_2 in equilibrium with it (ε_w) can be calculated as[14]

$$\varepsilon_w(‰) = \frac{17604}{273.15+T_l} - 17.93\ , \qquad \text{(Eqn. 21)}$$

where T_l is leaf temperature in °C. The $\delta^{18}O$ of CO_2 in equilibrium with evaporative site water (δ_{ce}) can then be calculated as

$$\delta_{ce} = \delta_e(1+\varepsilon_w) + \varepsilon_w\ . \qquad \text{(Eqn. 22)}$$

Eqns (1) to (22) provide two things: an estimate of δ_c, which is sensitive to the assumed vapour pressure inside the leaf, through its link to c_i; and an independent estimate of δ_{ce}, based on measurements of the $\delta^{18}O$ of transpired water, in combination with Eqn (18). Using this set of equations, we iteratively solved for the intercellular vapour pressure, e_i, subject to the constraint δ_c=δ_{ce}.

The calculation of δ_c requires an estimate of g_{mc}, the conductance to CO_2 from the intercellular air spaces to the sites of carbonic anhydrase activity, which we assume to be at the chloroplast surface[15]. The value of g_{mc} is needed to calculate c_{cs} for Eqn (16). We guessed values of g_{mc} such that they resulted in estimates of e_i near to e_s (vapour pressure inside the leaf near to saturation) when the air vapour pressure deficit (D) was at the lowest measurement values. The g_{mc} chosen for all *P. edulis* trees was 1 mol m^{-2} s^{-1} bar^{-1}. This value was also chosen for four out of six of the *J. monosperma* trees sampled in 2012, whereas a value of 0.6 mol m^{-2} s^{-1} bar^{-1} fit our criterion better for two other *J. monosperma* sampled in 2012, and for all *J. monsperma* sampled in 2013. These values were assumed for leaf temperatures of 25°C, and in all cases, g_{mc} was assumed to have a temperature dependence described by the equation $g_{mc}=(-0.26+0.051T_l)g_{mc(25)}$ where $g_{mc(25)}$ is the g_{mc} normalised to a leaf temperature of 25°C and T_l is leaf temperature[16].

To demonstrate the impact of choosing different values for g_{mc}, we conducted a sensitivity analysis, in which we calculated e_i/e_s for values of g_{mc} twice those originally assigned and for values half those originally assigned. These estimates of e_i/e_s are shown in Supplementary Figure 1. From this figure, one can see that doubling the assigned g_{mc} shifted the range of e_i/e_s estimates up, and halving them shifted the range of e_i/e_s estimates down. However the shifts were not so large as to substantially alter our interpretation of the data or our conclusion of significant unsaturation of e_i at

moderate to high D. We conducted an analysis of the impact on our calculations of e_i/e_s of assuming no temperature dependence for g_{mc}[17]. Assuming g_{mc} to be insensitive to temperature causes the calculated e_i/e_s to decline more sharply with increasing D than when a temperature dependence is incorporated.

2. Potential alternative explanations for $\delta_i > \delta_{ce}$ under assumption of saturated e_i

We interpret the observation that δ_i became larger than δ_{ce} at moderate to high D, as shown in Figure 2 of the main text, as evidence of unsaturation of e_i. Are there other possible explanations for this pattern? Here we consider five potential alternative explanations: (1) insufficient carbonic anhydrase activity, such that the $\delta^{18}O$ of CO_2 at the chloroplast surface would not be completely equilibrated; (2) somewhat less ^{18}O-enriched water at the chloroplast surface than that at the evaporative sites due to a Peclet effect[6]; (3) a fractionation factor for static diffusion of $H_2{}^{18}O$ through the stomatal pore of 32‰ rather than 28‰[11-13]; (4) an error associated with neglecting cuticular conductance in the gas exchange calculations[18,19]; and (5) a bias in the measurement of T_l by the energy balance method.

If the efficacy of carbonic anhydrase in catalysing the hydration of CO_2 decreased with decreasing leaf water potential, the $\delta^{18}O$ of CO_2 at the chloroplast surface might not have been completely equilibrated with local water. However, if this were the case, the effective δ_{ce} would decrease, because the unequilibrated portion of the CO_2 would have $\delta^{18}O$ lower than that which had equilibrated with evaporative site water. In that case, the calculated value of δ_{ce} would be smaller, and negative values of δ_{ce}-δ_i would become more negative than those shown in Fig 1, rather than less negative. Thus, potential incomplete equilibration resulting from insufficient carbonic anhydrase activity cannot correct negative values of δ_{ce}-δ_i.

What if the water at the chloroplast surface were somewhat less enriched than that at the evaporative sites due to a Peclet effect[6]? In this case, the calculated δ_{ce} would also be lower, and negative values of δ_{ce}-δ_i would again become more negative than those shown in Fig 1, rather than less negative. Therefore, the Peclet effect also cannot explain why δ_{ce}-δ_i became negative as D increased.

An uncertainty that would push the δ_{ce} in the other direction is that involving ε_k, the diffusional fractionation for $H_2{}^{18}O$. There is debate in the literature as to whether the fractionation factor for static diffusion of $H_2{}^{18}O$ through stomata should be 28 or 32‰[11-13]. In our calculations, we used the value 28‰, as this has been preferred most recently[13]. Increasing ε_k by using the static diffusional fractionation of 32‰ would increase the calculated $\delta^{18}O$ of water at the evaporative sites

and would therefore increase δ_{ce}. However, the increase would be on the order of 2 to 3‰, which has little influence on the overall trend of δ_{ce}-δ_i; values still become negative at moderate to high *D* as shown in Supplementary Figure 2. Therefore, choosing ε_k of 32‰ instead of 28‰ also cannot correct negative values of δ_{ce}-δ_i at moderate to high *D*.

It has been suggested recently that neglecting to account for cuticular conductance leads to significant overestimates of c_i, because g_s is calculated from a higher transpiration rate than that which would have actually occurred through the stomata[18,19]. We can rule this out as an alternative explanation for δ_{ce}-δ_i being negative at moderate to high *D* based on two considerations. First, negative δ_{ce}-δ_i results from c_i estimates which are too low, rather than too high. That is, the error in c_i associated with assuming e_i/e_s=1 when e_i/e_s is in fact less than one results in a c_i error that goes in the opposite direction to that which results from neglecting cuticular conductance. Therefore including cuticular conductance in the gas exchange equations would make negative values of δ_{ce}-δ_i more negative, rather than less negative. Second, cuticular conductance was measured in both *J. monosperma* and *P. edulis* at our study site, yielding mean values of 0.8 mmol m^{-2} s^{-1} for both species (S. Sevanto, unpublished). In our dataset, estimates of g_s assuming e_i/e_s=1 averaged 78 mmol m^{-2} s^{-1} for *J. monosperma* and 38 mmol m^{-2} s^{-1} for *P. edulis*. Therefore, transpiration through the cuticle would have averaged only 1% of that through the stomata in *J. monosperma* and only 2% of that through the stomata in *P. edulis*, and corrections to gas exchange calculations would be proportionally small.

Another parameter that involves uncertainty, and which could impact estimates of δ_{ce}-δ_i, is leaf temperature, T_l. Saturation vapour pressure is an exponential function of temperature, and any bias in the assessment of T_l during gas exchange measurements would translate directly into errors in c_i and therefore δ_{ce}-δ_i. The impact of a systematic bias of plus or minus 1°C in T_l on calculations of δ_{ce}-δ_i is shown in Supplementary Figure 3. With T_l shifted up by 1°C across the dataset, δ_{ce}-δ_i becomes negative at lower *D* for both *J. monosperma* and *P. edulis*. Whereas, with T_l shifted down by 1°C, δ_{ce}-δ_i becomes negative at higher *D* for both species. However, even in this case, δ_{ce}-δ_i still becomes negative at *D* of about 2 kPa in *P. edulis* and 4 kPa in *J. monosperma*.

This sensitivity analysis demonstrates the importance of accurate measurements of T_l for examining whether e_i unsaturates under physiologically relevant conditions. Accurate determination of T_l can be experimentally challenging[20,21]. The Li-Cor conifer cuvette that we used estimates T_l by energy balance. To test the accuracy of the energy balance estimations of T_l, we used a thermal imaging camera (FLIR SC6700; FLIR Systems, Wilsonville, OR, USA). We placed foliage samples in the cuvette and allowed them to reach steady gas exchange rates. We then began to acquire a

thermal imaging video of the cuvette and quickly opened it. Each pixel within the video frames was recorded as the surface temperature of the objects within the camera field of view. We used the first frame of the video in which the foliage sample was fully visible to calculate the average leaf temperature at the time of cuvette opening (Supplementary Figure 4). Foliage pixels in the image were separated from pixels showing the cuvette or other background objects, and then averaged. We then compared this estimate of T_l with that from the energy balance calculation prior to cuvette opening. The two estimates compared favourably across the range of T_l used in the study for both *J. monosperma* and *P. edulis* (Supplementary Figure 5). The intercept of the relationship between T_l from energy balance and T_l from the camera did not differ significantly from zero (P=0.10, n=30), and the slope did not differ significantly from unity (P=0.57, n=30).

Our check on the accuracy of T_l determined by energy balance by the Li-Cor 6400 photosynthesis system using the thermal imaging video camera indicated that T_l determined by the former method was within 1°C of T_l determined by the latter. Therefore, it is unlikely that a systematic error in leaf temperature caused δ_{ce}-δ_i to turn negative as D increased. Thus, the most parsimonious explanation remains that of e_i becoming unsaturated with increasing D. As a point of reference, however, we re-analysed the dataset to show the errors in measurement of T_l that would be required to satisfy the constraint δ_c=δ_{ce}, assuming e_i/e_s remained at unity throughout the course of measurements. In this set of calculations, we fixed e_i/e_s at unity and then allowed leaf temperature to vary freely, which caused e_s to vary according to the relationship shown in Eqn (4). The result of this analysis is shown in Supplementary Figure 6. Under the assumption that e_i/e_s=1, if the mismatch between δ_c and δ_{ce} were wholly caused by errors in leaf temperature, it would require leaf temperature to have been over-estimated by up to 2.5°C in *J. monosperma* and by up to 4.1°C in *P. edulis*. In this case, the intercept of the relationship between T_l from energy balance and T_l required to satisfy δ_c=δ_{ce} differed significantly from zero (P=0.01, n=125); the slope did not differ significantly from unity (P=0.70, n=125).

Having conifers as the subject of our experiment provided an advantage in terms of constraining potential errors in leaf temperature as it was estimated from energy balance. This is because the characteristic dimension of conifer leaves is small, and leaf temperatures are therefore relatively closely coupled to air temperatures[22]. Therefore, so long as air temperature in the cuvette was measured accurately, it is unlikely that there were significant errors in energy balance estimates of leaf temperature. Our experimental system had a further advantage in that it was free of the leaf temperature measurement error that can result from thermocouple sensing of leaf temperature, in which the thermocouple actually measures a mixture of leaf and air temperature[21]. In summary, both

experimental (thermal imaging) and theoretical (energy balance) considerations argue in favour of our leaf temperature estimates being sufficiently accurate to allow unsaturation of e_i to be resolved in our dataset.

3. Calculation of e_i from $\delta^{13}C$

Simultaneous observations of carbon isotope discrimination ($\Delta^{13}C$) provided additional evidence that e_i became unsaturated at moderate to high D. In our experiment, unsaturation of e_i caused an increasing error in c_i with increasing D. This manifested in negative estimates of the mesophyll contribution (Δ_{gm}) to observed $\Delta^{13}C$ at moderate to high D (Supplementary Figure 7). A negative value of Δ_{gm} would lead to a negative estimate of g_m, the mesophyll conductance to CO_2 from the intercellular air spaces to the sites of carboxylation within the chloroplasts. Such negative values of g_m contradict basic diffusional theory. A comprehensive $\Delta^{13}C$ model[5,23] can also be solved for e_i, when constrained by the observed $\Delta^{13}C$, if a value of g_m is assumed. We performed this calculation by assuming that g_m=g_{mc}/1.5[15,24]. Estimates of relative humidity inside the leaf calculated from $\Delta^{13}C$ were in reasonable agreement with those estimated by the $\delta^{18}O$ method (Supplementary Figure 8).

Here we describe our method for estimating e_i from carbon isotope discrimination. The comprehensive model for ^{13}C discrimination, including ternary effects[5], is as follows:

$$\Delta_{com} = \frac{1}{1-t}\left[a_b\frac{c_a-c_s}{c_a} + a_s\frac{c_s-c_i}{c_a}\right] + \frac{1+t}{1-t}\left[a_m\frac{c_i-c_c}{c_a} + b\frac{c_c}{c_a} - \frac{\alpha_b}{\alpha_e}e\frac{\mathcal{R}_d}{A+\mathcal{R}_d}\frac{c_c-\Gamma^*}{c_a} - \frac{\alpha_b}{\alpha_f}f\frac{\Gamma^*}{c_a}\right] \quad \text{(Eqn. 23)}$$

where c_a, c_s, c_i and c_c (μmol mol^{-1}) are the CO_2 mole fractions in ambient air, at the leaf surface, in the leaf intercellular air spaces, and at the sites of carboxylation in the chloroplast, respectively; a_b, a_s, and a_m are the fractionation factors associated with diffusion through the boundary layer (2.8‰), the stomatal pore (4.4‰), and in water (1.8‰), respectively; b and f are fractionations by RuBisCO carboxylation (30‰), and during photorespiration (16‰), respectively. The fractionation during day respiration, e, was calculated as[25,26] $e = e_{Rd} + e^*$. The e_{Rd} was assumed to be 0‰ and e^* was calculated as $e^*=\delta_{a(13)}-\Delta_{obs}-\delta_{substrate}$, where $\delta_{a(13)}$ is the $\delta^{13}C$ of CO_2 in the leaf cuvette during measurements, Δ_{obs} is the observed discrimination against ^{13}C, and $\delta_{substrate}$ is the $\delta^{13}C$ of likely respiratory substrates (-27.2‰ for *Juniperus monosperma* and -23.9‰ for *Pinus edulis*, calculated from the average observed discrimination for each species and assuming a $\delta^{13}C$ for ambient air of -8‰). The terms α_b, α_e, and α_f are 1+b, 1+e and 1+f, respectively. The A and $\mathcal{R}_d$ are the photosynthetic and day respiration rates (μmol m^{-2} s^{-1}), respectively. The Γ^* is the CO_2

compensation point in the absence of day respiration (μmol mol^{-1}). The t represents a ternary correction factor, calculated with Eqn (15), but with $\bar{a}$ for the calculation of α_{tc} (=1+$\bar{a}$) calculated using in Eqn (14) the fractionation factors for $^{13}CO_2$ rather than $C^{18}OO$.

Eqn (23) includes variables that are determined by gas exchange (c_a, c_i, A), fractionation factors that are assumed known (a_b, a_s, a_m, b, e, f, α_b, α_e, α_f) and several additional variables, calculated as follows:

- $c_s = c_a - \frac{1.37A}{g_b}$, where g_b is the boundary layer conductance to water vapour (mol m^{-2} s^{-1}), assumed known for the gas exchange cuvette.
- $c_c = c_i - \frac{A}{g_m P}$, where g_m is mesophyll conductance to CO_2 to the sites of carboxylation in chloroplasts (mol m^{-2} s^{-1} bar^{-1}) and P (bar) is the pressure of air surrounding the leaf.
- $\mathcal{R}_d = \mathcal{R}_{d\text{ at }25°C} \cdot 2^{\frac{T_l - 25}{10}}$, with $\mathcal{R}_{d\text{ at }25°C}$ assumed equal to 2 μmol CO_2 m^{-2} s^{-1}.
- $\Gamma^* = e^{13.49 - \frac{24460}{RT_k}}$, where R is the ideal gas constant (8.314 J K^{-1} mol^{-1}) and T_k is the leaf temperature in Kelvins[27].

The ^{13}C discrimination can also be measured (observed discrimination; Δ_{obs}), as described in Eqn (9), but with δ values for $\delta^{13}C$ rather than $\delta^{18}O$. The fractionation associated with the diffusion of CO_2 from the intercellular air spaces into the chloroplast (Δ_{gm}) can be calculated as[16]:

$$\Delta_{gm} = \Delta_i - \Delta_{obs} - \Delta_e - \Delta_f \ , \quad \text{(Eqn. 24)}$$

where Δ_i, Δ_e, and Δ_f are as follows:

$$\Delta_i = \frac{1}{1-t}\bar{a} + \frac{1}{1-t}[(1+t)b - \bar{a}]\frac{c_i}{c_a}, \quad \text{(Eqn. 25)}$$

$$\Delta_e = \frac{1+t}{1-t}\frac{\alpha_b}{\alpha_e} e \frac{\mathcal{R}_d}{A+\mathcal{R}_d}\frac{c_i - \Gamma^*}{c_a}, \quad \text{(Eqn. 26)}$$

$$\Delta_f = \frac{1+t}{1-t}\frac{\alpha_b}{\alpha_f} f \frac{\Gamma^*}{c_a}. \quad \text{(Eqn. 27)}$$

The Δ_{gm} can also be calculated as,

$$\Delta_{gm} = \frac{1+t}{1-t}\left[b - a_m - \frac{\alpha_b}{\alpha_e} e \frac{\mathcal{R}_d}{A+\mathcal{R}_d}\right]\frac{A}{g_m c_a}. \quad \text{(Eqn. 28)}$$

When we calculated Δ_{gm} with Eqn (24) we obtained negative values (Supplemental Figure 7). Because in Eqn (28) the term $\frac{1+t}{1-t}\left[b - a_m - \frac{\alpha_b}{\alpha_e} e \frac{\mathcal{R}_d}{A+\mathcal{R}_d}\right]\frac{A}{c_a} > 0$, a negative Δ_{gm} would lead to a negative estimate of g_m, which is nonsensical.

Alternatively, the difference between modelled ^{13}C discrimination could be reconciled by allowing e_i to differ from saturation. This can be achieved by solving Δ_{com} (Eqn. 23) for e_i, when constrained by Δ_{obs}, and assuming a value for g_m.

Because t depends on c_i through g_{tc}, solving Eqn (23) for c_i results in a quadratic equation with the following solution:

$$c_i = \frac{-II \pm \sqrt{II^2 - 4 \cdot I \cdot III}}{2 \cdot I}, \qquad \text{(Eqn. 29)}$$

where

$$I = a_s \cdot \left(\frac{e\mathcal{R}_d}{A+\mathcal{R}_d} - b - 1\right), \qquad \text{(Eqn. 30)}$$

$$II = \Delta_{com} c_a(-2 - a_s) + a_b(c_a - c_s) - a_s \left[\frac{A}{g_m P_a}\left(a_m - b + \frac{e\mathcal{R}_d}{A+\mathcal{R}_d}\right) + \Gamma^* \left(\frac{e\mathcal{R}_d}{A+\mathcal{R}_d} - f\right) + c_a - c_s + \frac{2A}{E}\right] + \left[c_a(2 + a_b) + c_s(a_s - a_b) + \frac{2A}{E}\right]\left[b - \frac{e\mathcal{R}_d}{A+\mathcal{R}_d}\right], \qquad \text{(Eqn. 31)}$$

$$III = [a_b(c_a - c_s) + a_s c_s]\left[c_a + \frac{2A}{E}\right] - \Delta_{com} c_a \left[\frac{2A}{E} - a_b c_a - c_s(a_s - a_b)\right] + \left[c_a(2 + a_b) + c_s(a_s - a_b) + \frac{2A}{E}\right]\left[\frac{A}{g_m P_a}\left(a_m - b + \frac{e\mathcal{R}_d}{A+\mathcal{R}_d}\right) + \Gamma^* \left(\frac{e\mathcal{R}_d}{A+\mathcal{R}_d} - f\right)\right]. \qquad \text{(Eqn. 32)}$$

The c_i can be calculated by substituting Δ_{obs} for Δ_{com}. Using this calculated c_i, the g_{tc}, g_s, and g_t can be calculated from Eqns (8), (7) and (2), respectively, and e_i solved for using Eqn (1).

References

1. von Caemmerer, S. & Farquhar, G. D. Some relationships between the biochemistry of photosynthesis and the gas exchange of leaves. *Planta* **153**, 376-387 (1981).
2. Evans, J. R., Sharkey, T. D., Berry, J. A. & Farquhar, G. D. Carbon isotope discrimination measured concurrently with gas exchange to investigate CO_2 diffusion in leaves of higher plants. *Aust. J. Plant Physiol.* **13**, 281-292 (1986).
3. Farquhar, G. D. *et al.* Vegetation effects on the isotope composition of oxygen in atmospheric CO_2. *Nature* **363**, 439-443 (1993).
4. Cernusak, L. A., Farquhar, G. D., Wong, S. C. & Stuart-Williams, H. Measurement and interpretation of the oxygen isotope composition of carbon dioxide respired by leaves in the dark. *Plant Physiol.* **136**, 3350-3363 (2004).
5. Farquhar, G. D. & Cernusak, L. A. Ternary effects on the gas exchange of isotopologues of carbon dioxide. *Plant Cell Environ.* **35**, 1221-1231 (2012).
6. Farquhar, G. D. & Lloyd, J. in *Stable Isotopes and Plant Carbon-Water Relations* (eds J.R. Ehleringer, A.E. Hall, & G.D. Farquhar) 47-70 (Academic Press, 1993).

7. Dubbert, M., Cuntz, M., Piayda, A. & Werner, C. Oxygen isotope signatures of transpired water vapor: the role of isotopic non-steady-state transpiration under natural conditions. *New Phyt.* **203**, 1242-1252 (2014).
8. Farquhar, G. D., Cernusak, L. A. & Barnes, B. Heavy water fractionation during transpiration. *Plant Physiol.* **143**, 11-18 (2007).
9. Bottinga, Y. & Craig, H. Oxygen isotope fractionation between CO_2 and water, and the isotopic composition of marine atmospheric CO_2. *Earth Planet. Sci. Lett.* **5**, 285-295 (1969).
10. Farquhar, G. D., Hubick, K. T., Condon, A. G. & Richards, R. A. in *Stable Isotopes in Ecological Research* (eds P. W. Rundel, J.R. Ehleringer, & K.A. Nagy) 21-46 (Springer-Verlag, 1989).
11. Merlivat, L. Molecular diffusivities of $H_2{}^{16}O$, $HD^{16}O$, and $H_2{}^{18}O$ in gases. *J. Chem. Phys.* **69**, 2864-2871 (1978).
12. Cappa, C. D., Hendricks, M. B., DePaulo, D. J. & Cohen, R. C. Isotopic fractionation of water during evaporation. *J. Geophys. Res.* **108**, 4525 (2003).
13. Luz, B., Barkan, E., Yam, R. & Shemesh, A. Fractionation of oxygen and hydrogen isotopes in evaporating water. *Geochim. Cosmochim. Acta* **73**, 6697-6703 (2009).
14. Brenninkmeijer, C., Kraft, P. & Mook, W. Oxygen isotope fractionation between CO_2 and H_2O. *Isot. Geosci.* **1**, 181-190 (1983).
15. Gillon, J. S. & Yakir, D. Internal conductance to CO_2 diffusion and $C^{18}OO$ discrimination in C_3 leaves. *Plant Physiol.* **123**, 201-213 (2000).
16. Evans, J. R. & von Caemmerer, S. Temperature response of carbon isotope discrimination and mesophyll conductance in tobacco. *Plant Cell Environ.* **36**, 745-756 (2013).
17. von Caemmerer, S. & Evans, J. R. Temperature responses of mesophyll conductance differ greatly between species. *Plant Cell Environ.* **38**, 629-637 (2015).
18. Boyer, J. S. Turgor and the transport of CO_2 and water across the cuticle (epidermis) of leaves. *J. Exp. Bot.* **66**, 2625-2633 (2015).
19. Hanson, D. T., Stutz, S. S. & Boyer, J. S. Why small fluxes matter: the case and approaches for improving measurements of photosynthesis and (photo)respiration. *J. Exp. Bot.* **67**, 3027-3039 (2016).
20. Morrow, P. A. & Slatyer, R. O. Leaf temperature effects on measurements of diffusive resistance to water vapor transfer. *Plant Physiol.* **47**, 559-& (1971).
21. Mott, K. A. & Peak, D. Alternative perspective on the control of transpiration by radiation. *Proc. Natl. Acad. Sci. USA* **108**, 19820-19823 (2011).

22. Campbell, G. S. & Norman, J. M. *An Introduction to Environmental Biophysics*. (Springer-Verlag, 1998).

23. Ubierna, N. & Farquhar, G. D. Advances in measurements and models of photosynthetic carbon isotope discrimination in C_3 plants. *Plant Cell Environ.* **37**, 1494-1498 (2014).

24. Barbour, M. M., Evans, J. R., Simonin, K. A. & von Caemmerer, S. Online CO_2 and H_2O oxygen isotope fractionation allows estimation of mesophyll conductance in C_4 plants, and reveals that mesophyll conductance decreases as leaves age in both C_4 and C_3 plants. *New Phyt.* **210**, 875-889 (2016).

25. Cernusak, L. A. *et al.* Environmental and physiological determinants of carbon isotope discrimination in terrestrial plants. *New Phyt.* **200**, 950-965 (2013).

26. Wingate, L., Seibt, U., Moncrieff, J. B., Jarvis, P. G. & Lloyd, J. Variations in ^{13}C discrimination during CO_2 exchange by *Picea sitchensis* branches in the field. *Plant Cell Environ.* **30**, 600-616 (2007).

27. Bernacchi, C. J., Pimentel, C. & Long, S. P. In vivo temperature response functions of parameters required to model RuBP-limited photosynthesis. *Plant Cell Environ.* **26**, 1419-1430 (2003).

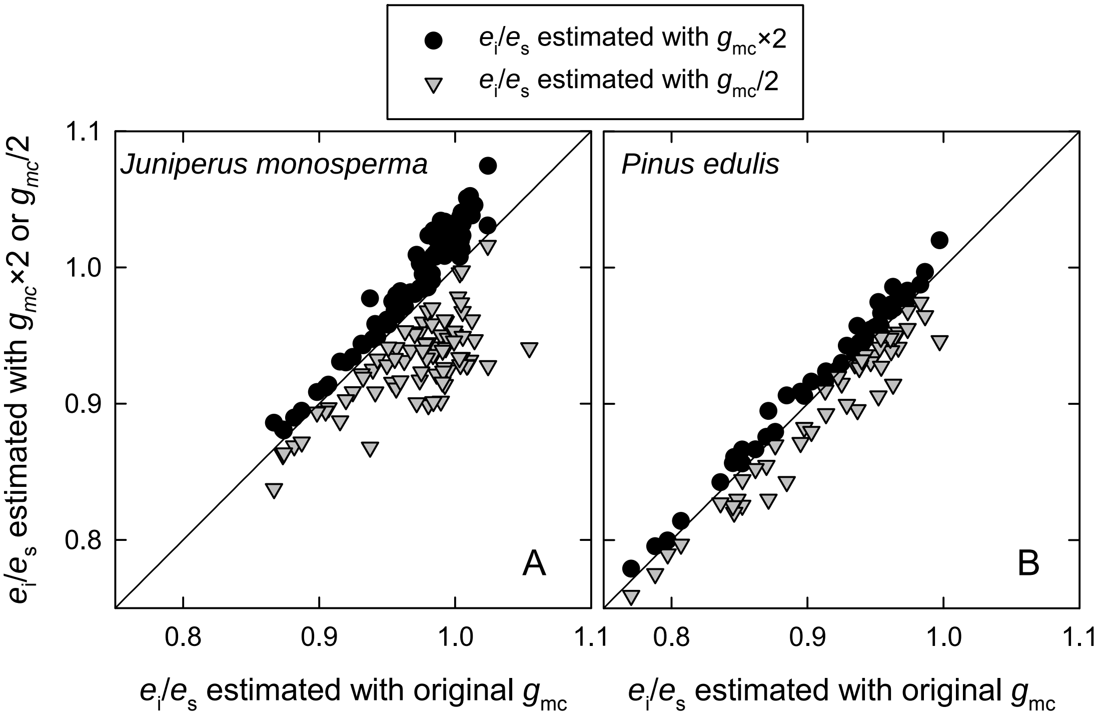

Supplementary Figure 1. A sensitivity analysis showing the effect of doubling or halving the original estimate of g_{mc} on the calculated relative humidity in the intercellular air spaces (e_i/e_s). The g_{mc} is the conductance to CO_2 from the intercellular air space to the site of carbonic anhydrase activity, assumed to be at the chloroplast surface. The original g_{mc} was chosen such that e_i/e_s would fall near unity at low air vapour pressure deficits (*D*). Panel A refers to *J. monosperma* and panel B to *P. edulis*.

Estimates of e_i/e_s were generally less sensitive to varying g_{mc} for *P. edulis* than for *J. monosperma*. This is because g_s was lower in *P. edulis* than in *J. monosperma*; mean values across the dataset were 0.04 and 0.08 mol H_2O m^{-2} s^{-1}, respectively. On the other hand, the initially assigned g_{mc} was similar, with mean values of 1.00 and 0.98 mol CO_2 m^{-2} s^{-1}, respectively. Thus the proportioning of the total resistance to CO_2 diffusion from the air to the chloroplast surface into stomatal and mesophyll components differed between the two species. In the case of *P. edulis*, the mesophyll resistance was a smaller proportion of the total resistance, and as a result, estimates of e_i/e_s were less sensitive to a doubling or halving of g_{mc}.

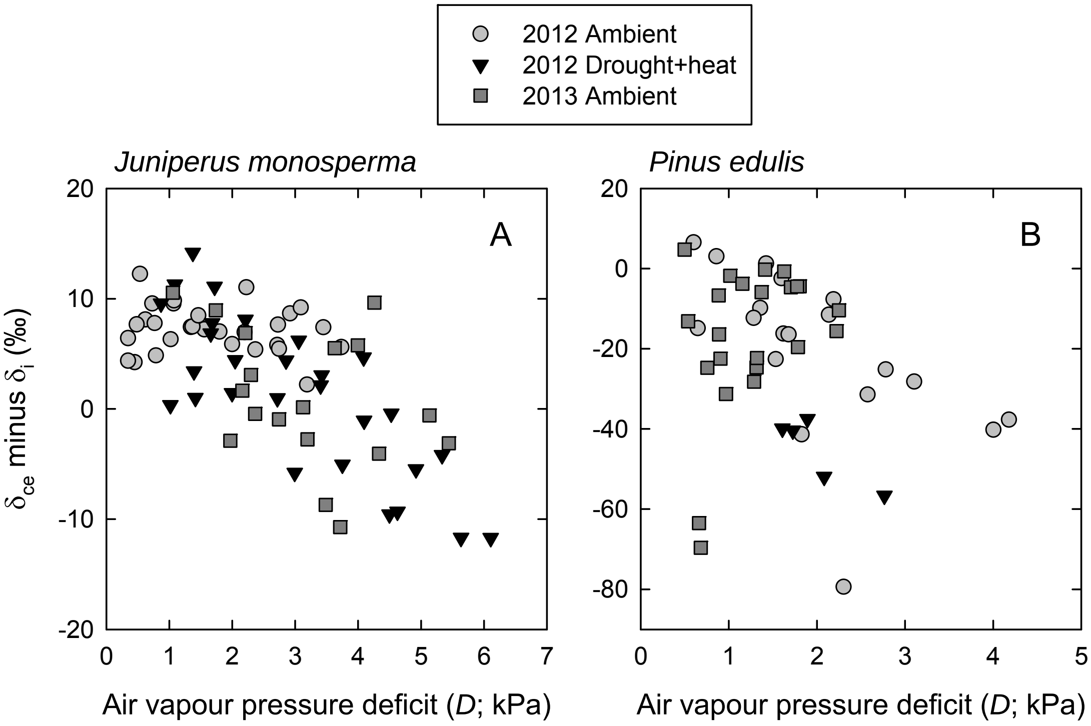

Supplementary Figure 2. Calculations of δ_{ce}-δ_i with the diffusional fractionation (ε_k) for $H_2{}^{18}O$ calculated with a fractionation factor for static diffusion of 32‰[12]. These results can be compared with those in Figure 1 of the main text, in which ε_k was calculated with a fractionation factor for static diffusion of 28‰[11]. Using 32‰ instead of 28‰ causes the values of δ_{ce}-δ_i to shift up by 2 to 3‰, such that overall, they become negative at slightly higher air vapour pressure deficits. Panel A refers to *J. monosperma* and panel B to *P. edulis*.

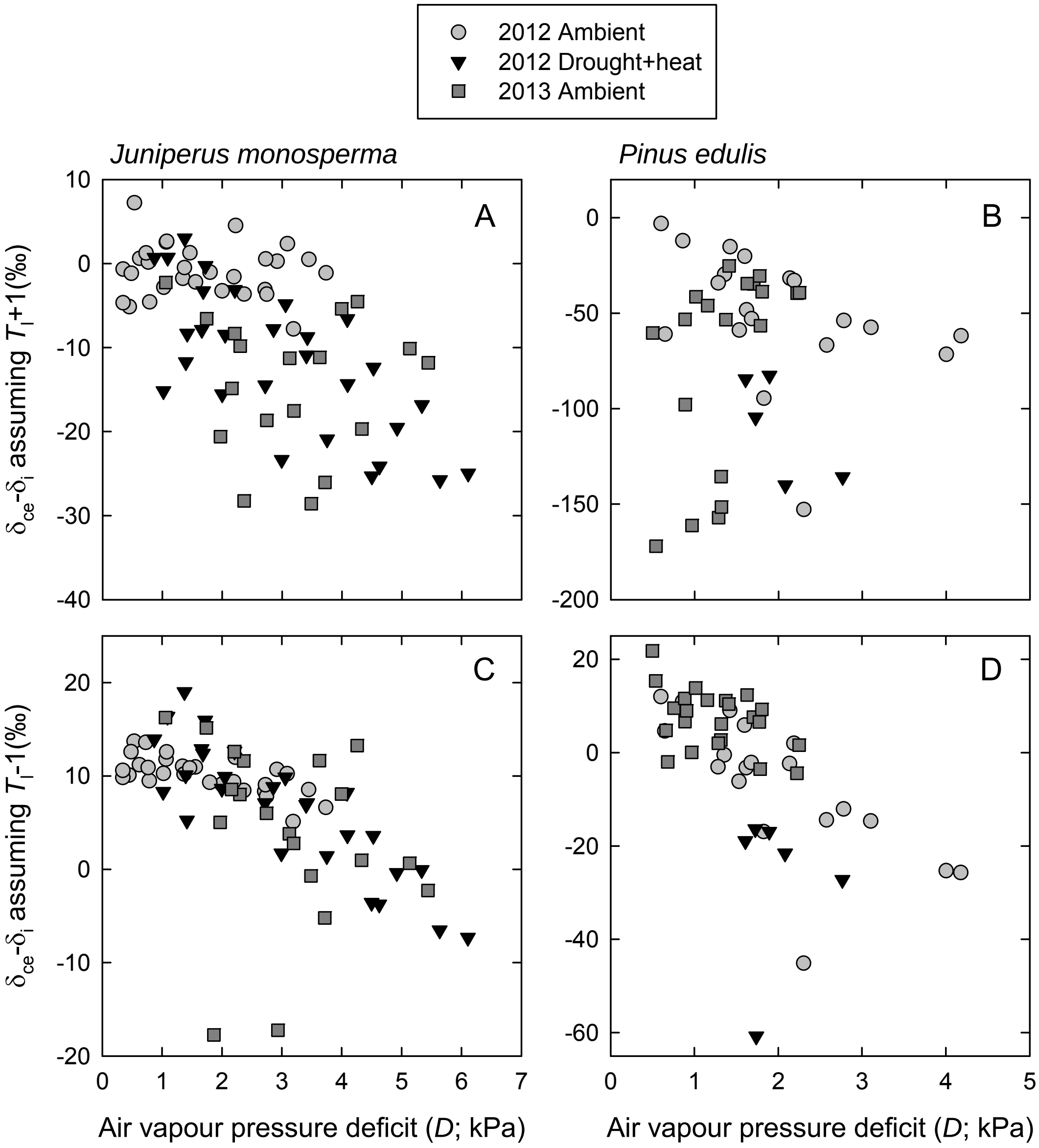

Supplementary Figure 3. A sensitivity analysis demonstrating the effect on calculated values of δ_{ce}-δ_i of increasing or decreasing leaf temperature (T_l) by 1°C. Increasing T_l by 1°C (A, B) increases the saturation vapour pressure, thereby exaggerating the extent of unsaturation of intercellular vapour pressure, e_i. This causes δ_{ce}-δ_i to become negative at lower air vapour pressure deficits. Decreasing T_l by 1°C (C, D) reduces the extent of unsaturation of e_i, thereby shifting δ_{ce}-δ_i up to higher values, such that it becomes negative at higher air vapour pressure deficits. Panels A and C refer to *J. monosperma* and panels B and D to *P. edulis*.

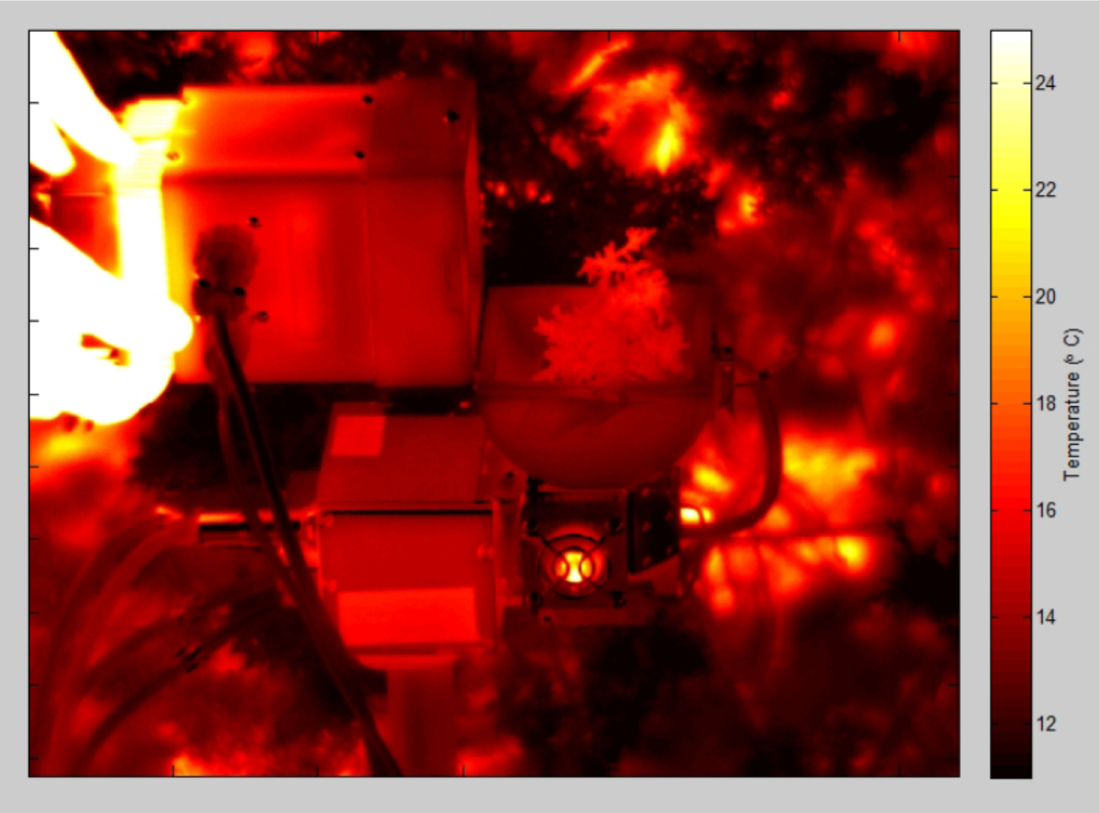

Supplementary Figure 4. A thermal image of the foliage inside the gas exchange cuvette just after it was opened. Video was acquired of the cuvette being opened with a thermal imaging camera and the first frame in which the foliage was completely visible was used to calculate the average leaf temperature. This average leaf temperature was then compared to the average leaf temperature estimated by energy balance in the Li-Cor portable photosynthesis system prior to opening the cuvette.

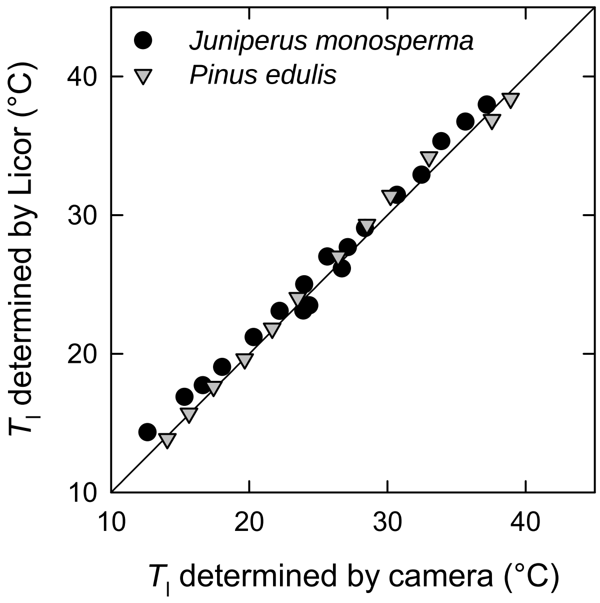

Supplementary Figure 5. A comparison of the average leaf temperature (T_l) determined by energy balance inside the Li-Cor conifer leaf cuvette with the T_l determined with a thermal imaging video camera immediately upon opening the cuvette. The one-to-one line is shown inside the panel.

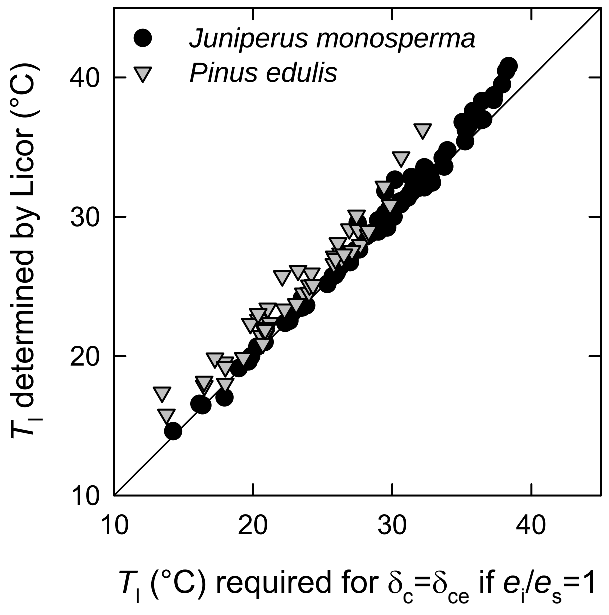

Supplementary Figure 6. A comparison of leaf temperature (T_l) determined by energy balance inside the Li-Cor conifer leaf cuvette with the T_l that would be required to satisfy the condition that $\delta_c=\delta_{ce}$ if e_i/e_s were fixed at unity. In this case e_s (and therefore e_i) varies according to the exponential relationship between e_s and T_l. The T_l on both axes is in °C and the one-to-one line is shown inside the panel.

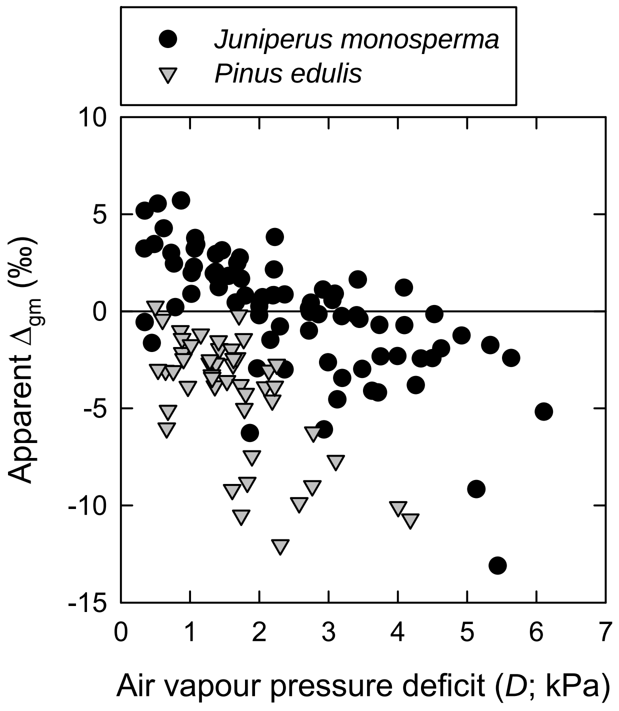

Supplementary Figure 7. The residual component of carbon isotope discrimination ($\Delta^{13}C$) attributed to mesophyll conductance (Δ_{gm}) plotted against the air vapour pressure deficit (*D*) to which the leaves were exposed within the gas exchange cuvette. The Δ_{gm} is the residual between observed and modelled $\Delta^{13}C$, where the modelled $\Delta^{13}C$ in this case includes all known fractionations except the mesophyll component. The modelled $\Delta^{13}C$ was calculated with gas exchange parameters assuming e_i/e_s=1. Negative values of Δ_{gm} are not theoretically possible and indicate that the assumption of e_i was not valid at moderate to high air vapour pressure deficits.

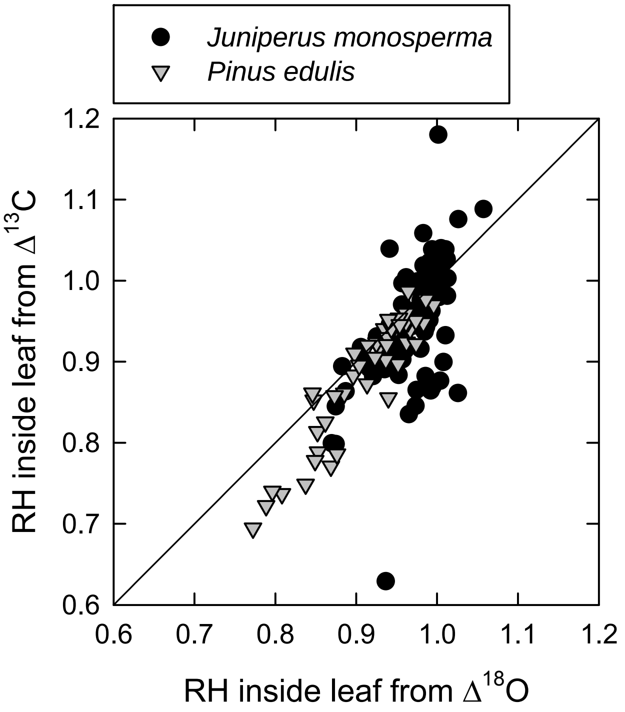

Supplementary Figure 8. Relative humidity in the intercellular air spaces (e_i/e_s) estimated from observations of carbon isotope discrimination ($\Delta^{13}C$) in CO_2 passing over the leaf plotted against that estimated from observations of oxygen isotope discrimination ($\Delta^{18}O$) in CO_2 and water vapour passing over the leaf. The method using $\Delta^{13}C$ requires more assumptions than that using $\Delta^{18}O$, and we therefore consider the $\Delta^{18}O$ method more robust. Nevertheless, the two show reasonable agreement and both indicate unsaturation of the intercellular vapour pressure, e_i. The diagonal line is the one-to-one line.
